# Requirement of the 3′-UTR-dependent suppression of DAZL in oocytes for pre-implantation mouse development

**DOI:** 10.1371/journal.pgen.1007436

**Published:** 2018-06-08

**Authors:** Kurumi Fukuda, Aki Masuda, Takuma Naka, Atsushi Suzuki, Yuzuru Kato, Yumiko Saga

**Affiliations:** 1 Division of Mammalian Development, Genetic Strains Research Center, National Institute of Genetics, Mishima, Shizuoka, Japan; 2 Department of Genetics, SOKENDAI, Mishima, Shizuoka, Japan; 3 Division of Materials Science and Chemical Engineering, Graduate School of Engineering, Faculty of Engineering, Yokohama National University, Yokohama Kanagawa, Japan; 4 Department of Biological Science, Graduate School of Science, The University of Tokyo, Bunkyo-ku, Tokyo, Japan; University of Pennsylvania, UNITED STATES

## Abstract

Functional oocytes are produced through complex molecular and cellular processes. In particular, the contribution of post-transcriptional gene regulation mediated by RNA-binding proteins (RBPs) is crucial for controlling proper gene expression during this process. DAZL (deleted in azoospermia-like) is one of the RBPs required for the sexual differentiation of primordial germ cells and for the progression of meiosis in ovulated oocytes. However, the involvement of DAZL in the development of follicular oocytes is still unknown. Here, we show that *Dazl* is translationally suppressed in a 3′-UTR-dependent manner in follicular oocytes, and this suppression is required for normal pre-implantation development. We found that suppression of DAZL occurred in postnatal oocytes concomitant with the formation of primordial follicles, whereas *Dazl* mRNA was continuously expressed throughout oocyte development, raising the possibility that DAZL is dispensable for the survival and growth of follicular oocytes. Indeed, follicular oocyte-specific knockout of *Dazl* resulted in the production of normal number of pups. On the other hand, genetically modified female mice that overexpress DAZL produced fewer numbers of pups than the control due to defective pre-implantation development. Our data suggest that post-transcriptional suppression of DAZL in oocytes is an important mechanism controlling gene expression in the development of functional oocytes.

## Author summary

Evolutionarily conserved DAZ family genes are indispensably involved in germline development. *Dazl* (*deleted in azoospermia-like*) is a member of the mammalian DAZ family of genes, and plays crucial roles in the sexual differentiation of primordial germ cells and spermatogenesis, and is implicated in the progression of meiosis II in ovulated eggs. Despite its importance for multiple processes during germline development, its participation in follicular oocyte development is enigmatic. This study addressed this issue and found that DAZL is translationally suppressed in postnatal oocytes in a 3′-UTR-dependent manner. Furthermore, this suppression is required for normal pre-implantation development after fertilization, suggesting the presence of an unidentified mechanism controlling DAZL expression. Our data provide new insights for post-transcriptional gene regulation involved in oocyte development.

## Introduction

The production of functional oocytes is an essential process in the female ovary, by which genetic information is continuously passed to the next generations. For successful oocyte development, gene expression needs to be precisely regulated according to the developmental stages and environmental cues such as gonadotropic hormones. Oocytes that fail to regulate proper gene expression are degenerated during their development or are unable to proceed with embryonic cleavage even if they are fully developed [1]. Therefore, unveiling the mechanisms controlling the quality of oocytes is a crucial issue to understand the molecular basis of female reproduction.

Post-transcriptional gene regulation mediated by RNA-binding proteins is an important molecular mechanism involved in this process. An evolutionarily conserved and well-documented post-transcriptional event is the translational suppression and storage of maternal mRNAs with shorter poly (A) tails [2]. Although the genome is actively transcribed and proteins are produced during oocyte growth, transcription become inactive in full-grown oocytes and maternal mRNAs are used for protein synthesis in early zygote development [3]. These processes are orchestrated by a battery of relevant RNA-binding proteins, including cytoplasmic polyadenylation element binding proteins (CPEB), maskin, and other germ cell-specific RNA-binding proteins [4,5,6,7]. In addition to the maturation process, germ cell-specific RNA-binding proteins are also responsible for multiple processes in oogenesis. For instance, CPEB1 and Pumilio1 are involved the progression of meiotic prophase I in the embryonic ovary[8,9], and MSY2 is involved in follicle development after birth in mice [10,11], suggesting the significant contribution of post-transcriptional gene regulation throughout oogenesis.

Deleted in azoospermia-like (DAZL) is a member of the evolutionarily conserved DAZ family of RNA-binding proteins that acts as a translational activator in mice [12]. Biochemical and structural analyses showed that DAZL binds to the U-rich region of its target’s 3′-UTR [13,14,15], and genetic analyses revealed that DAZL is indispensable for gametogenesis in both males and females [16,17,18]. As DAZL is reportedly required for sexual differentiation of primordial germ cells, progression of meiotic prophase I in embryonic female germ cells [19], and for the progression of meiosis in maturating oocytes [20], it is believed that DAZL is involved in female germ cell development throughout oogenesis. However, the role of DAZL in follicular oocytes remains unknown because *Dazl*-deficient oocytes die due to the failure of meiotic progression in the embryonic ovary [16,21]. Moreover, although the previous immunohistochemical analysis demonstrated that DAZL was expressed in both embryonic and follicular oocytes in postnatal ovaries [16], it was also noted that DAZL signals were not detectable by western blotting in ovaries 1 to 2 weeks after birth [22]. Therefore, further analysis is required to clarify this contradiction.

In this study, we investigated DAZL expression in embryonic and postnatal ovaries, and found that DAZL was translationally suppressed in a 3′-UTR-dependent manner in follicular oocytes. Genetic analysis by knocking out the *Dazl* gene in a follicular oocyte-specific manner indicated that *Dazl* is dispensable for follicular growth, maturation, and fertilization. On the other hand, the 3′-UTR-dependent suppression of DAZL in follicular oocytes is required for the progression of normal pre-implantation development. Our data clarify the previously ambiguous expression pattern of DAZL in the postnatal ovary, and simultaneously demonstrate the significance of the post-transcriptional suppression of DAZL in follicular oocytes.

## Results

### DAZL is post-transcriptionally suppressed in postnatal oocytes

**Fig 1:**
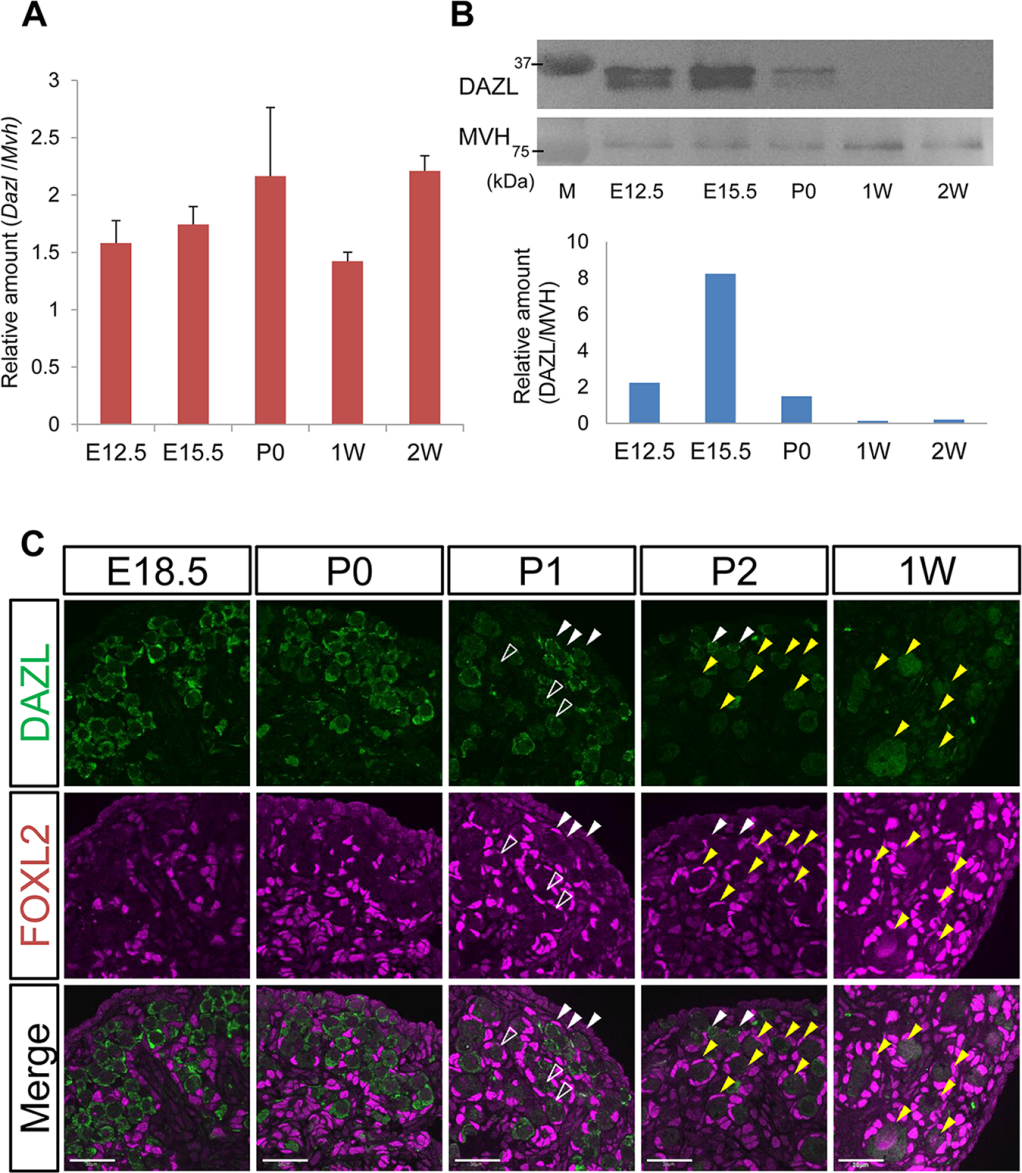
DAZL expression is suppressed in postnatal oocytes. (**A**) RT-qPCR analysis for *Dazl* in wild-type (WT) female gonads at E12.5 (n = 4), E15.5 (n = 3), P0 (n = 3), 1 week (1W) (n = 3), and 2 weeks (2W) (n = 3). *Mvh* (also known as *Ddx4*) was used as a normalizer because this gene is continuously expressed in germ cells from E12.5 to secondary follicles [24]. The vertical axis represents relative expression level of *Dazl* to *Mvh*. Error bars represent S.D. (**B**) Western blotting analysis for DAZL from E12.5 to 2W ovaries. Two bands representing DAZL were detected. As the molecular weight of DAZL is estimated to be 33KDa, the lower band corresponds with this. However, both bands disappear in *Dazl* knockout ovaries [25], indicating that both two bands are DAZL signals. Quantification of western data is shown below. The vertical axis represents relative DAZL expression level normalized by MVH. (**C**) Immunofluorescence analysis for DAZL (green) and FOXL2 (magenta) from E18.5 to P2, and 1W ovaries. White arrowheads indicate cystic oocytes, and yellow arrowheads indicate primordial follicles. Open arrowheads indicate oocytes showing weaker DAZL expression. Scale bar, 30μm.

To examine *Dazl/*DAZL expression in detail, we performed quantitative reverse transcription-polymerase chain reaction (RT-qPCR) and western blotting analyses using ovaries from embryos until juvenile stages (Fig 1A and 1B). RT-qPCR data showed that *Dazl* mRNA was constantly expressed and expression differences were less than 2-fold among all stages investigated (Fig 1A). On the other hand, DAZL expression was markedly changed during oocyte development (Fig 1B). Although it was abundant in embryonic ovaries, with the strongest expression at embryonic day (E) 15.5, the expression declined in newborn ovaries. Afterwards, DAZL expression further declined and was hardly detectable in 1- and 2-week ovaries, which is consistent with previous descriptions [22].

As DAZL expression was significantly decreased in the newborn ovary and onward, we next asked whether this reduction in DAZL was correlated with the formation of primordial follicles. In the embryonic ovary, oocytes are connected with each other by intercellular bridges (ICBs). Within a few days after birth, ICBs are broken and each oocyte is enclosed by pre-granulosa cells, resulting in the formation of primordial follicles [23]. Thus, we examined the expression changes of DAZL in perinatal ovaries by immunostaining DAZL together with a granulosa cell marker, forkhead box protein L2 (FOXL2), from E18.5 to 1W ovaries. Strong DAZL signals were observed in most oocytes until the day of birth (P0), when a large number of oocytes were still connected with each other. However, at one day after birth (P1), its expression began to decrease in some oocytes (Fig 1C, open arrowheads). The expression of DAZL was further decreased at two days after birth (P2), at which point, many oocytes formed primordial follicles (Fig 1C, yellow arrowheads) and exhibited weaker expression than cystic-oocytes (Fig 1C, white arrowheads). Thereafter, the weakened DAZL expression was observed in 1-week ovaries. These data suggest that DAZL is decreases in oocytes coinciding with the development of primordial follicles.

### DAZL is not required for oogenesis in the postnatal ovary

**Fig 2:**
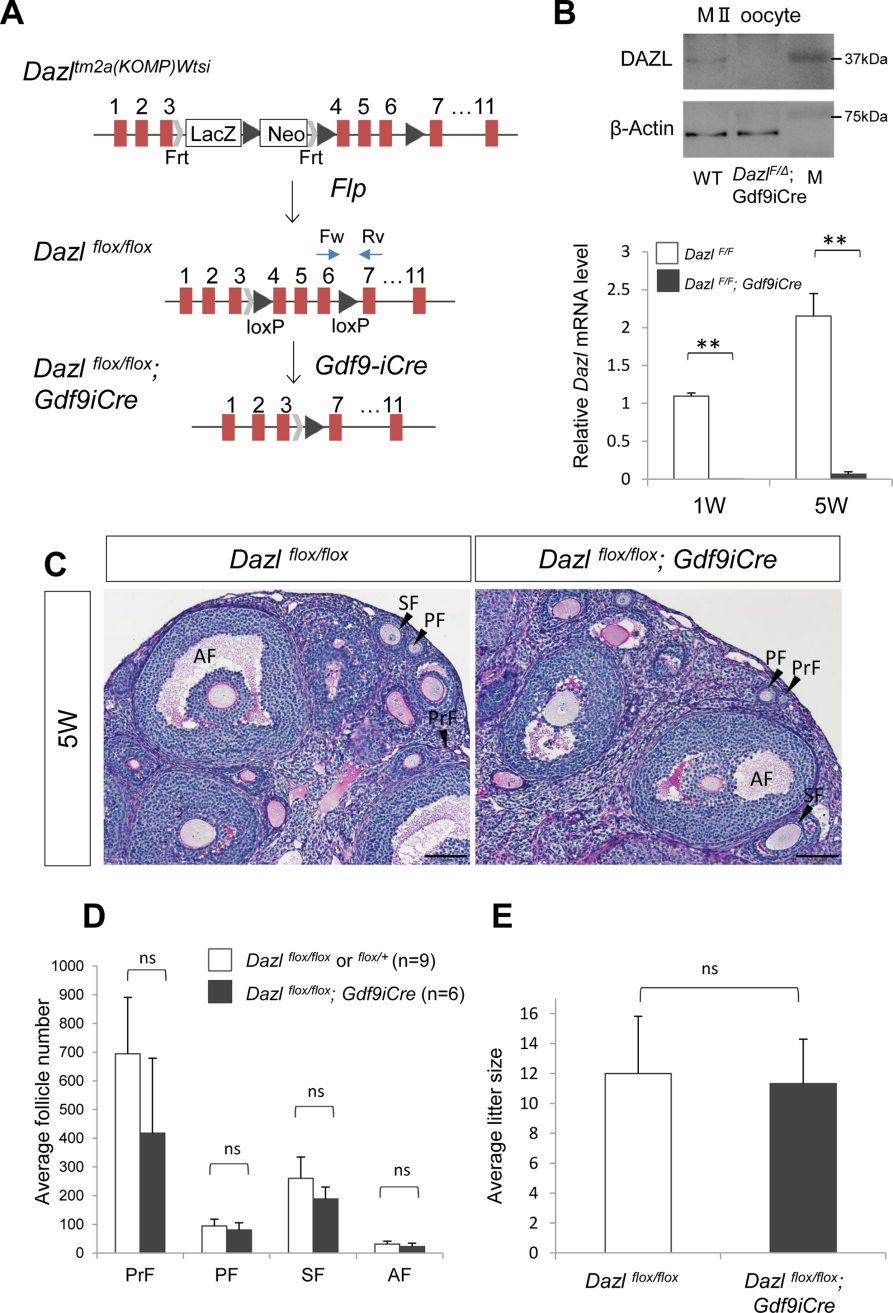
Postnatal oocyte-specific *Dazl* knock-out mice do not exhibit any defects. (**A**) A schematic diagram of the generation of conditional *Dazl* knockout mice. *Dazl* ^*flox/flox*^ females were crossed with *Dazl* ^*flox/flox*^*;Gdf9-iCre* male mice to obtain *Dazl*^*flox/flox*^*;Gdf9-iCre* (conditional *Dazl* knockout) females. Blue arrows indicate the position of primers used for RT-qPCR in (B). (**B**) Western blotting analysis for WT and *Dazl* cKO MII oocytes (upper panels), and RT-qPCR analysis for *Dazl* in 1 and 5W ovaries using a primer set that amplifies only the WT allele (lower graph, n = 3). Error bars represents S.D. Significance level of changes are indicated (two-tailed student's t-test; ** *P* < 0.005). (**C**) Periodic acid-Schiff (PAS) staining of control (left) and cKO (right) ovaries at 5W after birth. PrF, primordial follicle; PF, primary follicle; SF, secondary follicle; and AF, antral follicle. Scale bars, 100μm. (**D**) Follicle counting analysis of control (white bar, n = 9) and *Dazl* cKO (black bar, n = 6) ovaries at 5 weeks after birth. Error bars represents S.D. ns, no significant difference between control and *Dazl* cKO ovaries (two-tailed student's t-test). (**E**) Average litter size of control (n = 3) and *Dazl* cKO females (n = 5). Error bars represent S.D. ns, same as in (**D**).

Both immunostaining and western blotting analyses revealed that DAZL was decreased in oocytes shortly after birth, which raised the possibility that DAZL is dispensable for follicular development. To test this possibility, we used conditional *Dazl* knockout (cKO) mice (Fig 2A). *Dazl*^*flox*^ mice were crossed with a postnatal oocyte-specific Cre mouse line, *Gdf9-iCre*, which expresses improved Cre recombinase from P2 oocytes [25]. The *Dazl* gene was successfully disrupted by *Gdf9*-*iCre*, as evidenced by RT-qPCR and western blotting, in which both *Dazl* mRNA and DAZL protein were hardly detectable in *Dazl* cKO ovaries (Fig 2B). We also confirmed that our *Dazl* KO (*Dazl* ^*1lox/1lox*^) mouse line recapitulated the phenotype of previous *Dazl* knockout females (S1 Fig)[16]. On histological analysis, *Dazl* cKO ovaries as well as control ovaries contained both primordial and growing follicles (Fig 2C). Notably, cKO ovaries did not have any significant differences in the number of primordial or growing follicles (Fig 2D). These data suggest that DAZL is dispensable for the survival and growth of follicular oocytes.

In order to evaluate the reproductive capability of *Dazl* cKO oocytes, we next crossed *Dazl* cKO females with wild-type (WT) males. We found that *Dazl* cKO females were fertile and produced a normal number of pups (Fig 2E). The average litter size delivered from *Dazl* cKO females (11.3±0.62) was almost identical with that from WT (12.0±2.83). We also confirmed that all progeny delivered by *Dazl* cKO females were heterozygotes for the *Dazl*^*1lox*^ allele (n = 258). These results were surprising because a previous report stated that *Dazl* knockdown in MII oocytes results in the defective progression of the oocyte to zygote transition [20]. However, MII oocytes derived from *Dazl* cKO females did not have abnormal spindle morphology (S2 Fig). These data indicate that DAZL is not required for the maturation of oocytes or subsequent fertilization.

### DAZL is suppressed in a 3′-UTR dependent manner

**Fig 3:**
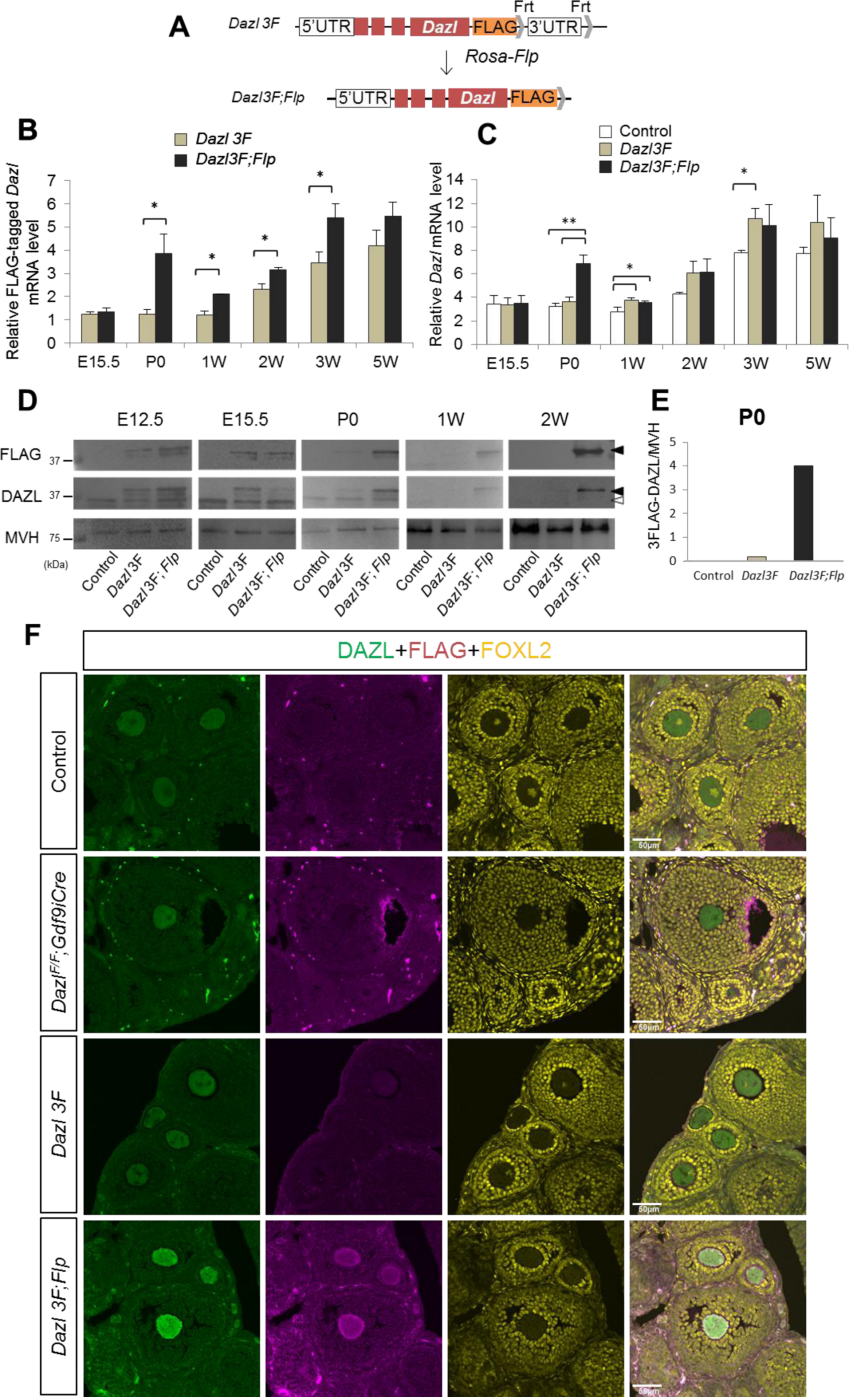
3’-UTR-dependent suppression of DAZL in follicular oocytes. (**A**) Schematic diagram of the bacterial artificial chromosome (BAC)-carrying transgenic mouse line. The *Dazl*'s 3′-UTR was removed by crossing with *Rosa-Flp* hetero male mice. (**B**) RT-qPCR for *3Flag-Dazl* using ovary extracts from E15.5 to 5 weeks (n = 3, except for *Dazl 3F;Flp* at E15.5, n = 4). The vertical axis represents the relative mRNA expression level of *3Flag-Dazl* normalized by *Mvh*. Significance level of changes are indicated (two-tailed student's t-test; *p < 0.05). (**C**) RT-qPCR for total (endogenous and 3Flag) *Dazl* from E15.5 (control, n = 8; *Dazl* 3F, n = 3; *Dazl 3F;Flp*, n = 4), P0 (n = 3), 1W (n = 4; 3; 3), 2W (n = 3; 3; 4), and 5W (n = 3) ovaries. The vertical axis represents relative mRNA expression level of total *Dazl* normalized by *Mvh*. Significance level of changes are indicated (two-tailed student's t-test; **P<0.005, *P<0.05). (**D**) Western blotting analysis of FLAG (top) and endogenous DAZL (middle). MVH was used as a control. Filled and open arrowheads indicate FLAG- and endogenous DAZL, respectively. (**E**) Relative quantification of FLAG-DAZL between control (white bar), *Dazl 3F* (gray bar), and *Dazl 3F;Flp* (black bar) in P0 ovary. The vertical axis represents the normalized values of FLAG-DAZL by MVH. (**F**) Immunostaining of 3W ovary from control, *Dazl* cKO, *Dazl 3F* and *Dazl 3F;Flp* using antibodies against for DAZL (green), FLAG (magenta), and FOXL2 (yellow). Scale bars, 50μm.

In embryonic male germ cells, *Dazl* is post-transcriptionally suppressed in a 3′-UTR -dependent manner by a male-specific RNA-binding protein, NANOS2 [26]. As DAZL decreases in postnatal ovaries, it is possible that *Dazl* is also post-transcriptionally suppressed in a 3′-UTR -dependent manner by unidentified mechanisms in follicular oocytes. In order to test this possibility, we used our bacterial artificial chromosome (BAC)-carrying transgenic mouse line, in which the FLAG tag was inserted at the C-terminus of *Dazl* and the *Dazl* 3′-UTR was flanked with *Frt* sequences (*Dazl 3F*, Fig 3A upper) [26]. The significance of the *Dazl* 3′-UTR for its expression was assessed by crossing the BAC transgenic female with a *Rosa-Flp* male (*Dazl 3F;Flp*, Fig 3A lower). RT-qPCR showed that the amount of *Flag*-*Dazl* mRNA was increased in *Dazl 3F;Flp* ovaries after birth (Fig 3B). However, the effect of removing the 3′-UTR was not clear because the difference in *Flag-Dazl* mRNA expression levels between *Dazl 3F* and *Dazl 3F;Flp* was less than 2-fold, and the total *Dazl* expression level (*Flag-Dazl* +endogenous *Dazl*) was not changed between *Dazl 3F* and *Dazl 3F;Flp* except in the P0 ovary (Fig 3B and 3C). In contrast to the small increase in the mRNA level, FLAG-DAZL expression was greatly increased after birth (Fig 3D). Although FLAG-DAZL (filled arrowheads) decreased in *Dazl 3F* ovaries from P0 onward, which was consistent with the reduction in endogenous DAZL (open arrowhead), its expression was continuously observed in P0, 1W, and 2W ovaries when the 3′-UTR was removed. Quantification of FLAG-DAZL expression revealed that its expression increased 20-fold in *Dazl 3F;Flp* at P0 (Fig 3E). The results of western blotting were also supported by immunostaining. Both total- and FLAG-DAZL expression was strongly observed in *Dazl 3F;Flp* ovaries (Fig 3F and S3 Fig), whereas their expression levels in WT and *Dazl 3F* ovaries were comparable with those in *Dazl* cKO ovaries. Furthermore, strong DAZL expression was observed in all stages of follicular oocytes in *Dazl 3F;Flp* ovaries (S3 Fig). These data indicate that DAZL is post-transcriptionally suppressed in a 3′-UTR-dependent manner in follicular oocytes.

### Role of DAZL suppression in female reproduction

**Fig 4:**
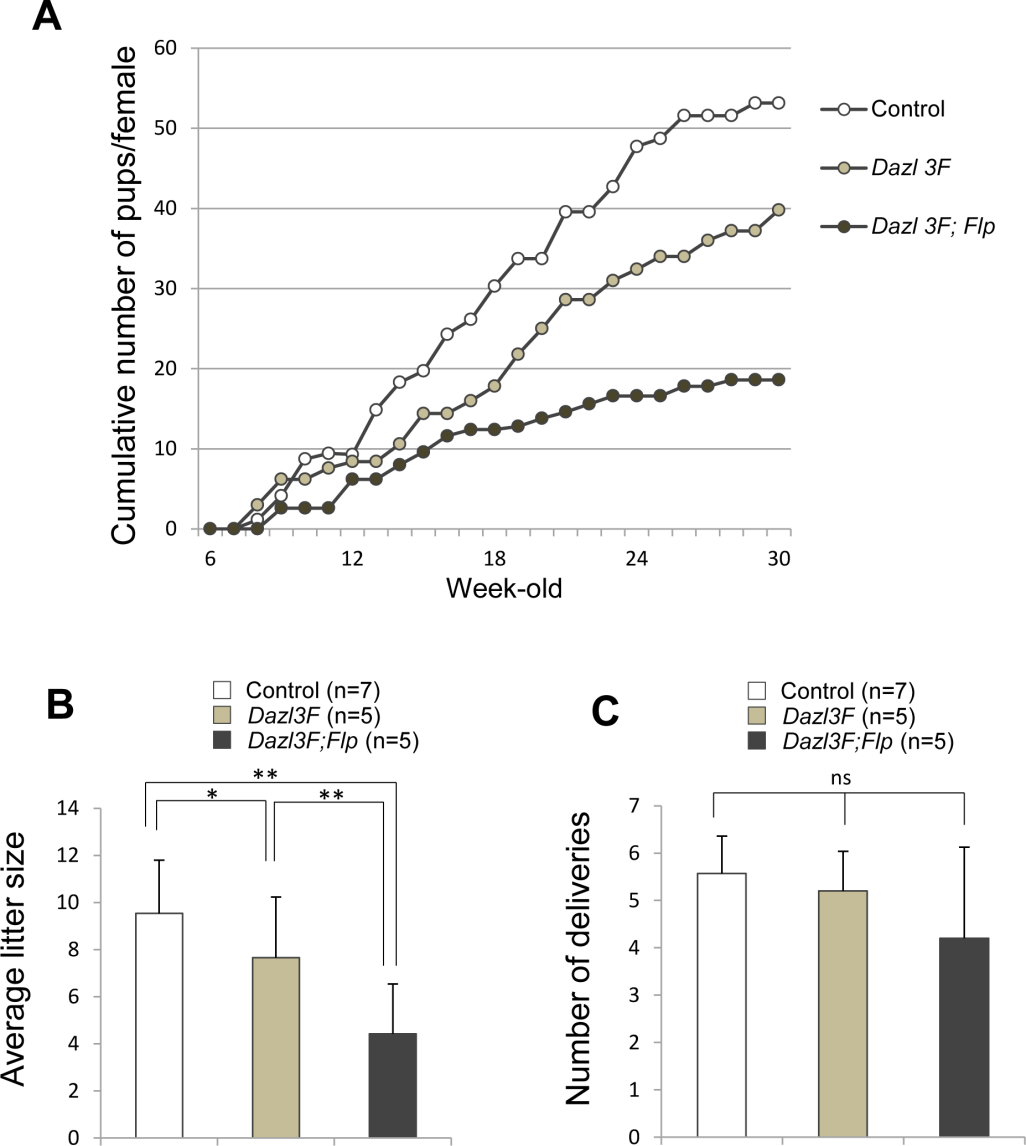
DAZL overexpression induces litter size reduction. (**A**) Litter size analysis of control (n = 7), *Dazl 3F* (n = 5), and *Dazl 3F;Flp* (n = 5) females. Each female was crossed with WT males from 6 to 30W. Vertical axis represents the average cumulative number of pups. (**B**) Averaged litter size for control (white bar), *Dazl 3F* (gray bar), and *Dazl 3F;Flp* (black bar). Error bars, S.D. Significance level of changes are indicated (Tukey HSD test; *P<0.05, **P<0.005). (**C**) Averaged number of deliveries in control (white bar), *Dazl 3F* (gray bar), and *Dazl 3F;Flp* (black bar). Error bars, S.D. ns, no significant differences among control, *Dazl 3F*, and *Dazl 3F;Flp* (Tukey HSD test).

To investigate the role of 3′-UTR-dependent DAZL suppression in female reproduction, we crossed BAC transgenic females with WT males when female mice reached 6 weeks old. Each pair was kept in a breeding cage until female mice became 30 weeks old, and the number of pups delivered during this period was counted. We found that BAC transgenic females were fertile regardless of the presence or absence of the *Dazl* 3′-UTR (Fig 4A). The number of total pups was slightly lower by in *Dazl3F* females (39.8±5.5, n = 5) compared with control females (53.1±9.1, n = 7). Interestingly, *Dazl 3F;Flp* females produced less than half the normal number of pups (18.6±11.3, n = 5). As 3FLAG-DAZL protein rescued the germless phenotype in *Dazl*^*-/-*^ mice [26], it is unlikely that the observed litter size reduction was caused by the expression of 3FLAG-DAZL. Thus, these results suggest that DAZL overexpression results in litter size reduction. We next analyzed the number of deliveries and the number of pups in each delivery. The number of pups in each delivery was fewer by *Dazl 3F* and *Dazl 3F;Flp* mice (Fig 4B), but the number of deliveries was not significantly different among genotypes (Fig 4C). These results suggest that the reduced female fecundity was due to defects during follicular development, fertilization, or zygote development after fertilization, but not to the shortened reproductive lifespan.

### Excess DAZL is deleterious for pre-implantation development

**Fig 5:**
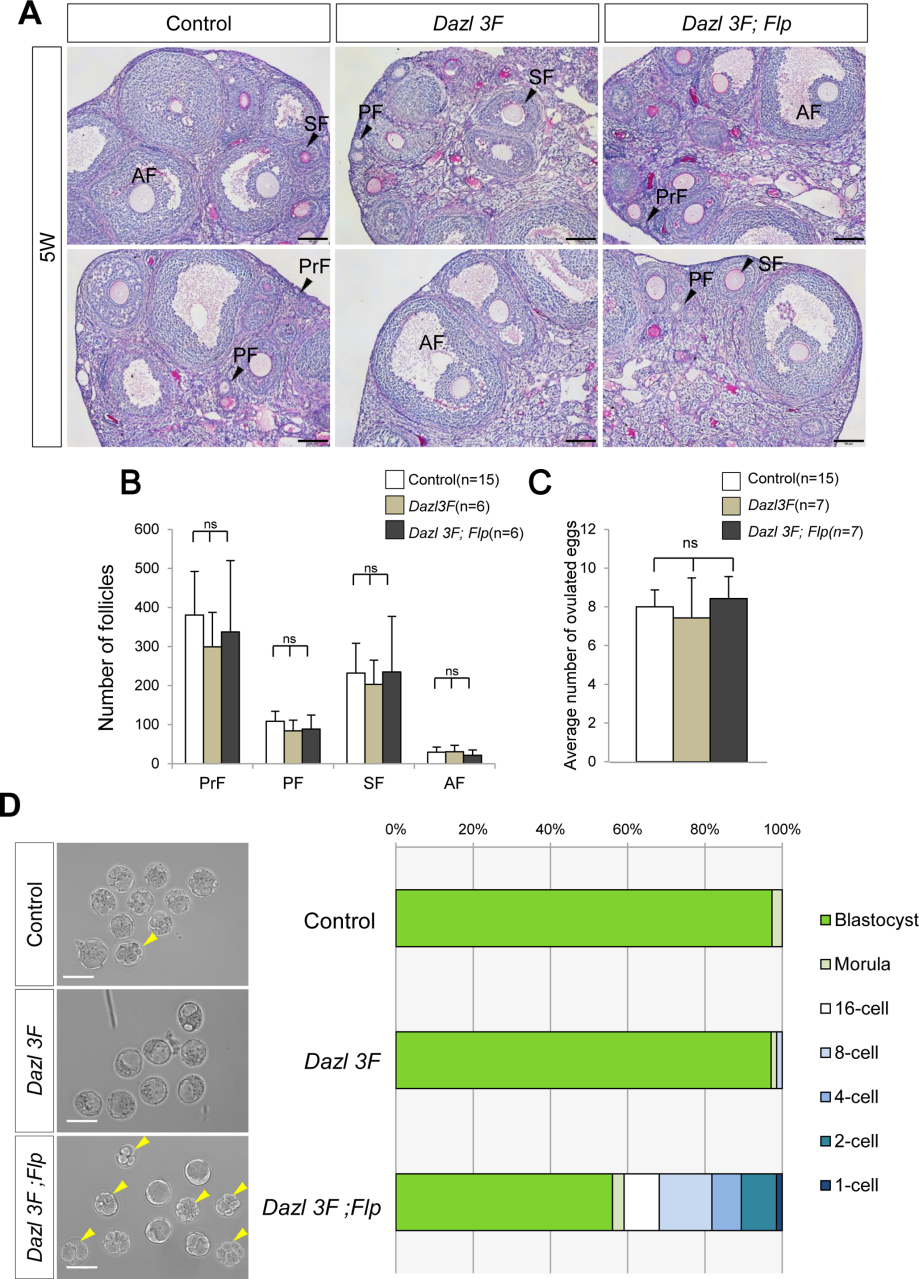
Defective pre-implantation development is a cause of the litter size reduction. (**A**) PAS staining of control, *Dazl* 3F, and *Dazl 3F;Flp* ovaries at 5 weeks after birth. PrF, PF, SF, and AF are same as in Fig 2C. Scale bars, 100μm. (**B**) Follicle counting analysis for control (n = 15), *Dazl 3F* (n = 6), and *Dazl 3F;Flp* (n = 6) using ovarian sections at 5 weeks after birth. Error bars represent S.D. ns, no significant difference among control, *Dazl 3F*, and *Dazl 3F;Flp*. (**C**) The average number of ovulated eggs from control (n = 15), *Dazl 3F* (n = 7), and *Dazl 3F;Flp* (n = 7) females. Error bars, S.D. ns, no significant difference among control, *Dazl 3F*, and *Dazl 3F;Flp* (Tukey HSD test). (**D**) Analysis of pre-implantation development in BAC transgenic females. (Left) E3.5 embryos from control, *Dazl 3F*, and *Dazl 3F;Flp* females. Yellow arrowheads indicate abnormal embryos. Scale bars, 100μm. (Right) Proportion of embryos that developed to each stage up to blastocysts. Embryos collected from pregnant females at E3.5 were counted. Delayed embryos were cultured for an additional two days and ones that reached the blastocyst stage were added. Control (n = 75), *Dazl 3F* (n = 69) and *Dazl 3F;Flp* (n = 56).

To determine the cause of the litter size reduction in the DAZL overexpressing females, we examined the development of oocytes, fertilization, and pre-implantation development. Histological analysis revealed that BAC transgenic ovaries did not have significantly different numbers of primordial, primary, secondary or antral follicles compared with WT ovaries (Fig 5A and 5B). We next asked whether ovulation normally occurs by counting the number of one-cell embryos ovulated. However, the number was not significantly different among the genotypes (Fig 5C). These data suggest that folliculogenesis and subsequent ovulation proceeds normally even in BAC transgenic females. Thus, to examine whether these ovulated eggs developed normally, we measured the proportion of blastocysts by flushing E3.5 embryos from oviducts. We cultured the collected embryos for a further two days and then counted the embryos because the different timing of sexual behavior in each mouse pair influences the progression of early embryonic development (Fig 5D). We found that only 56.1% of embryos derived from *Dazl 3F;Flp* females developed into blastocysts, whereas more than 97.3 and 97.1% embryos derived from control and *Dazl 3F* females became blastocysts, respectively. The development of the remaining 43.9% of *Dazl 3F;Flp* embryos stopped at the 1-cell to morula stages. These observations were reproduced in 1-cell culture experiments, in which development was specifically disrupted in embryos from *Dazl 3F;Flp* females (S4A Fig). Statistical analysis revealed that development was arrested during 1- to 4-cell and 8-cell to blastocyst stages in embryos from *Dazl3F;Flp* mother (S4B Fig). Furthermore, the spindle morphology was normal in *Dazl3F;Flp* oocytes (S2 Fig). These results indicate that the reduction of pups in *Dazl 3F;Flp* females was due to defective pre-implantation development. As strong DAZL expression was observed in *Dazl 3F;Flp* until the MII oocyte stage but decreased in 1-cell embryos and was no longer detectable in 2-cell embryos (S5 Fig), it is likely that abnormal expression of DAZL in oocytes causes the defective pre-implantation development.

## Discussion

In this study, we demonstrated that DAZL expression is post-transcriptionally suppressed in a 3′-UTR-dependent manner in postnatal oocytes. Although DAZL has been thought to function in postnatal oocytes, our data suggest that DAZL is not required for postnatal oocyte development. Supporting this idea, analysis of conditional *Dazl* knockout mice revealed that DAZL is dispensable for postnatal oocyte development. Furthermore, excess DAZL expression results in litter size reduction. These data indicate that post-transcriptional regulation of *Dazl* plays a crucial role in normal female reproduction.

It was previously reported that DAZL was expressed in growing oocytes [16], but a later study stated that DAZL was not detectable in the postnatal ovary [22]. Therefore, it has been unclear whether DAZL plays a role in follicular oocytes. Our results answered this question; DAZL expression is suppressed in follicular oocytes and is dispensable for oogenesis after birth. Interestingly, this suppression coincides with the formation of primordial follicles. As *Dazl* mRNA was continuously expressed in oocytes regardless of developmental stage, it is likely that post-transcriptional gene regulatory mechanisms are altered between cystic oocytes and follicular oocytes. Importantly, DAZL suppression requires its 3′-UTR, suggesting the presence of some mechanisms regulating DAZL expression in postnatal oocytes. In general, post-transcriptional regulation is conducted by microRNAs and RNA-binding proteins [27]. However, it was reported that the function of microRNA is globally suppressed in oocytes and early embryonic development [28]. Thus, it is possible that *Dazl* expression is regulated by some RNA-binding proteins (RBPs). One possible candidate RBP for DAZL suppression is CPEB1, a mammalian ortholog of *Xenopus* CPEB. CPEB acts as both a translational activator and suppressor of its target mRNAs depending on its phosphorylation state [29,30]. CPEB1 is expressed in postnatal oocytes and promotes the translation of *Dazl* in MII oocytes[20], thus it may suppress *Dazl* in follicular oocytes. Further expression and functional analyses, including the phosphorylation state, of CPEB1 are required to address this question.

Our oocyte-specific *Dazl* KO females exhibited no ovarian developmental defect and the MII oocytes had no spindle abnormalities. Furthermore, the *Dazl* cKO females produced normal numbers of pups. These observations were inconsistent with Chen and colleague’s results that DAZL depletion in MII oocytes results in defective spindle formation in meiosis II [20]. One possible explanation for this contradiction is the method of gene depletion. We used the Cre-loxP system for *Dazl* cKO *in vivo*, whereas Chen et al. used morpholino knock-down in MII oocytes. A recent zebrafish report found that approximately 80% of phenotypes induced by morpholino do not correlate with mutant phenotypes induced by ZFN, TALEN or CRISPER/Cas9; therefore, the above-mentioned knock-down phenotype may have emerged due to indirect effects [31]. Alternatively, it is possible that some system that compensates for DAZL function works in *Dazl*-cKO MII oocytes because a previous study reported that the activation of a compensation system rescued deleterious mutations, which was not observed after translational or transcriptional knockdown [32]. Further analysis is required to evaluate the contribution of RNA-binding proteins for the progression of meiosis II.

Although DAZL expression is suppressed after birth, introducing the BAC transgenic allele in the *Dazl*^*+/+*^ background reduced litter size even in the presence of the 3′-UTR (Fig 4). As a previous study reported that *Dazl* dosage in females influences their litter size, and *Dazl*^*+/−*^ females produced more pups than *Dazl*^*+/+*^ females [22], the slight reduction in litter size by our *Dazl* 3F mice may be attributed to the dosage effect. However, our histological and embryo culture experiments did not reveal any abnormalities in *Dazl 3F* mice. In addition, we were unable to observe obvious differences in resorption after implantation. One possible explanation is that insertion of the BAC transgene influences female reproduction.

Our results suggest that the suppression of *Dazl* translation in follicular oocytes is required for producing the proper number of progeny. However, why excess DAZL expression causes defective pre-implantation development remains still unclear. DAZL has been implicated in the positive regulation of translation [14,33,34], thus it is possible that the observed defect may be due to abnormal translational promotion. Alternatively, it is also possible that excess DAZL abnormally suppresses its target RNAs because DAZL works as a component of stress granules, cytoplasmic RNP granules involved in translational suppression or mRNA storage, in the testis [35,36]. Therefore, it is likely that suppression of DAZL expression in follicular oocytes is an important molecular mechanism for controlling proper gene expression.

## Materials and methods

### Ethics statement

All mouse experiments were approved by the Animal Experimentation Committee at the National Institute of Genetics (approval number 30–5) and Yokohama National University (approval number 2017–09) and conducted under the Regulations for Animal Experiments at the National Institute of Genetics, Research Organization of Information and Systems and the guideline at Yokohama National University.

### Mice

Mice were housed in a specific-pathogen-free animal care facility at the National Institute of Genetics (NIG). All experiments were approved by the NIG Institutional Animal Care and Use Committee and the animal experimental committee at Yokohama National University. The genetic background of mice used in this study was C57BL/6N (Clea Japan), except in the DAZL expression analysis and conditional *Dazl* knockout mice (mixed genetic background of ICR and C57BL/6N). The BAC-carrying transgenic mouse line was generated in a previous study [26]. The BAC transgenic mice were backcrossed with C57BL/6N at least 3 times. *Dazl* flox mice were generated from an ES cell line produced by the Knock Out Mouse Project (KOMP, Dazl^tm2a (KOMP) Wtsi^).

### Reverse transcription-quantitative polymerase chain reaction (RT-qPCR)

Total RNAs were isolated from whole gonads of wild-type and BAC transgenic mice at each stage by RNeasy Mini Kit (Qiagen). One hundred ng (1W to 5W) and 40 ng (E12.5 to P0) of total RNA were used for cDNA synthesis using Prime Script RT Reagent Kits with gDNA Erase according to the manufacturer’s protocol (Takara). Real time PCR was performed with KAPA SYBR FAST qPCR kits using a thermal cycle dice real time system (Takara). The obtained data was normalized by *Mvh*.

The following primers were used for PCR amplification:

Dazl

Forward: 5′−CACGCCTCAGTGACTCGGCGAC−3’

Reverse: 5′−CGAAGCATACAGACAGTGGTC−3’

Mvh

Forward: 5′−GTTGAAGTATCTGGACATGATGCAC−3’

Reverse: 5′−CGAGTTGGTGCTACAATAATACACTC−3’

G3pdh

Forward: 5′−ACCACAGTCCATGCCATCAC−3’

Reverse: 5′−TCCACCACCCTGTTGCTGTA−3’

FLAG tagged *Dazl*

Forward: 5′−CACGCCTCAGTGACTCGGCGAC−3’

Reverse: 5′−CACCGTCATGGTCTTGTAGTC−3’

Dazl cKO

Forward: 5′−GACTTACATGCAGCCTCCAACCATG−3’

Reverse: 5′−AACAGGCAGCTGATATCCAGTGATG−3’

### Western blotting

Ovaries were lysed in RIPA buffer (50 mM Tris-HCl (pH8.0), 150 mM NaCl, 0.5% Sodium deoxycholate, 0.1% Sodium dodecyl sulfate, 1% NP-40) and sonicated. After removing the debris by centrifugation, lysates were dissolved in 2xSDS sample buffer, and heated. MII oocytes and 1-cell zygotes were lysed in 10μl 2xSDS sample buffer. Each sample was applied to gels for SDS-PAGE and transferred to nitrocellulose membranes. The membranes were blocked in 5% skim-milk in TBST (50mM Tris-HCl (pH7.5),150mM NaCl, 0.1% Tween-20) for 1 hour at room temperature (RT). Membranes were incubated with primary antibodies (Abcam, anti-rabbit DAZL antibody, 1:2000 for ovarian sample or 1:500 for MII, 1-cell and 2-cell zygote / Abcam, anti-rabbit DDX4 antibody, 1:1000/Santa Cruz, anti-mouse βactin, 1:2000/Sigma, anti-FLAG antibody,1:2000) diluted in 3% skim-milk in TBST or Can Get Signal immunoreaction Enhancer Solution (TOYOBO) overnight at 4°C. After washing the membranes with TBST, membranes were incubated with anti-rabbit HRP-conjugated secondary antibody (Cell signaling, 1:5000) and anti-mouse HRP-conjugated secondary antibody (Cell signaling, 1:5000) in TBST or Can Get Signal immunoreaction Enhancer Solution, respectively, at RT for 90 min. The signals were detected by SuperSignal West Femto Maximum Sensitivity Substrate (Thermo Scientific) and AE-9300H EZ-CAPTURE MG (ATTO). Western blotting results were quantified by Gel Analysis with ImageJ software.

### Immunostaining for paraffin embedded samples

Ovaries were fixed in 4% PFA (paraformaldehyde) at 4°C overnight and embedded in paraffin wax. Each sample was sliced at 6-μm thickness and placed on glass slides. After removing the paraffin wax and autoclaving in antigen unmasking solution/high pH (Vector Laboratories), glass slides were washed in PBST (PBS, 0.1%Tween-20) and pre-incubated in 3% skim milk in PBST blocking solution at RT for 1 hour. The slides were reacted with primary antibodies (Anti-DAZL antibody, Abcam, 1:200 / Anti-FOXL2 antibody, Abcam, 1:200/ Anti-FLAG antibody, SIGMA, 1:10000) at 4°C overnight. Then, slides were washed with PBST and incubated with second antibodies (Alexa 488 Donkey anti-Rabbit, Life technologies, 1:1000 /Alexa 594 Donkey anti-Mouse, Life technologies, 1:1000/Alexa 594 Donkey anti-Goat, Life technologies, 1:1000 / Cy5 Donkey anti-goat, Rockland, 1:1000) at RT for 60 min. DNA was counter-stained with DAPI, and fluorescent images were obtained using confocal microscopy FV1200 (Olympus).

### Immunostaining for frozen samples

Ovaries were fixed with 4% PFA (paraformaldehyde) at 4°C overnight, which was then graded to 30% sucrose, and ovaries were then embedded in O.C.T compound (Sakura Fine tek). Each sample was sliced at 6-μm thickness. After removing the O.C.T compound, slides were incubated with 3% skim milk in PBST (PBS, 0.1% Tween-20) for 1 hour. Primary antibody reactions were performed with the following dilutions (Anti-DAZL antibody, Abcam, 1:200 / Anti-FOXL2 antibody, Abcam, 1:200) at 4°C overnight. After washing with PBST, secondary antibody reaction was performed with the following dilutions (Alexa 488 Donkey anti-Rabbit, Life technologies, 1:400 /Alexa 594 Donkey anti-Goat, Life technologies, 1:400) at RT for 90 min. Slides then were counter-stained by DAPI at RT for 15 min. Fluorescent images were obtained by confocal microscopy FV1200 (Olympus).

### Immunostaining of MII oocytes

MII oocytes were fixed with MeOH at -20°C for 3 minutes, washed with PBS-TX (0.1%TritonX, PBS), and were then incubated with blocking solution (3%BSA, 0.1%TritonX, PBS) at 4 ^o^C for 3 hours. Primary antibody reactions were performed with the following dilutions (Anti-α-tubulin antibody, Sigma, 1:1000) at 4°C overnight. After washing with PBS-TX, secondary antibody reaction was performed with the following dilutions (Alexa 488 Donkey anti-Rabbit, Life technologies, 1:1000) and DAPI at RT for 60 min. Then, oocytes were washed with PBS-TX. Fluorescent images were obtained using confocal microscopy FV1200 (Olympus).

### Histological analysis

Histological analysis was carried out by PAS (Periodic acid-Schiff) staining according to the standard protocol. Briefly, ovaries were fixed in Bouin solution, embedded in paraffin wax, and sliced at 6-μm thickness. The sections were submerged in xylene, 100%, 90%, 70% ethanol, and distilled water at RT, and stained with PAS solution. Ovarian images were obtained with an inverted microscope BX 51 and 61(Olympus). Follicle stages were counted on every 5 sections.

### Litter size investigation

*Daz*l^+/+^, *Dazl 3F*, and *Dazl 3F;Flp* females at 6 weeks old were crossed with C57BL/6N males, and kept together until female mice reached 30 weeks old. The number of pups and deliveries was recorded. Pups were removed after counting the number and sex. Females that killed their pups were excluded from the analysis.

### Collection of MII oocytes, 1-cell, 2-cell embryos and blastocysts

To obtain MII, 1-cell and 2-cell oocytes for western blotting, female mice were injected with PMSG (ASKA Pharmaceutical). Forty-eight hours after PMSG injection, mice were stimulated with hCG (ASKA Pharmaceutical) for 14 h and MII oocytes were collected. To obtain western blotting samples of 1-cell and 2-cell embryos, each female was crossed with a WT male after hCG injection. Eggs with obvious abnormalities were removed from experiments. One-cell embryos for investigation of ovulation number and pre-implantation development investigation were obtained from the ampulla of pregnant females at E0.5. Blastocysts for examining progression of early embryonic development were obtained by flushing oviducts at E3.5. Collected blastocysts were cultured for two days in KSOM medium (Ark resource).

### Statistical analysis

Significance was assessed by the Student’s t-test for differences between two samples. For quantitative analyses among multiple samples, significance was assessed using one-way ANOVA followed by Tukey HSD (Honest Significant Difference) test. Asterisks in figures indicate significance: *P < 0.05, **P < 0.005.

## Supporting information

S1 Fig *Dazl*^*1lox/1lox*^ females recapitulate the *Dazl* knockout phenotype. (**A**) A photograph of *Dazl*^*1lox/+*^ and *Dazl*^*1lox/1lox*^ 3W ovaries. Scale bar, 1 mm. (**B**) PAS staining of *Dazl*^*1lox/+*^ and *Dazl*^*1lox/1lox*^ 3W ovaries. Note that homozygous mutants contain no oocytes, reminiscent of the previous *Dazl* knockout ovary [16]. PrF, PF, SF, and AF are the same as in Fig 2C. Scale bar, 100 μm.
(TIF)

S2 Fig Spindle morphology in *Dazl 3F; Flp* and *Dazl* cKO M II oocytes. Immunostaining of MII oocytes of control (n = 85), *Dazl*^*f/f*^*;Gdf9iCre* (n = 34), *Dazl 3F* (n = 45) and *Dazl 3F; Flp* (n = 37) MII oocytes using an antibody against for α-tubulin (green). DNA was counterstained with DAPI (magenta). Scale bar, 40 μm.
(TIF)

S3 Fig DAZL expression in postnatal oocytes. Immunostaining of 5W ovaries of control, *Dazl*^*f/f*^*;Gdf9iCre*, *Dazl 3F* and *Dazl 3F; Flp* mice. Antibodies against for DAZL (green) and FOXL2 (magenta) were used, and DNA was counterstained with DAPI. PrF, PF, SF, and AF are same as in Fig 2C. Scale bar, 50 μm.
(TIF)

S4 Fig Survival rate analysis from 1-cell zygote to blastocyst stage. **(A)** Survival rates of preimplantation embryos. One-cell stage zygotes (n = total number of embryos examined, number of used mothers) were collected from control (n = 120, 15), *Dazl 3F* (n = 59, 7) and *Dazl 3F; Flp* (n = 54, 7) mothers. The proportion of surviving zygotes at each stage was calculated as follows: the number of surviving zygotes out of the number of 1-cell zygotes in each experiment (mother). Error bars, S.D. Significance level of changes are indicated (Tukey HSD; **P<0.005, *P<0.05).
**(B)** Statistical analysis of surviving zygotes from 1-cell zygotes to blastocysts in *Dazl 3F; Flp* (n = 54). The p-value for the average number of zygotes in each stage was calculated using Tukey HSD. P<0.05 is written in red.
(TIF)

S5 Fig DAZL expression in preimplantation embryos. (**A**) Western blotting analysis of MII oocytes and 1- and 2-cell embryos. Both FLAG and endogenous DAZL were detected using the anti-DAZL antibody. Anti-β actin antibody was used as a loading control. Note that both FLAG and endogenous DAZL were not detectable in 2-cell embryos. Filled and open arrowheads indicate FLAG- and endogenous DAZL, respectively.
(**B**) Quantification of western blotting results for MII and 1-cell samples. The vertical axis represents relative DAZL expression level normalized by β actin.
(TIF)
