# Supplementary figures and images for: Requirement of the 3′-UTR-dependent suppression of DAZL in oocytes for pre-implantation mouse development
Source: PLoS Genet. 2018 Jun 8;14(6):e1007436. doi: 10.1371/journal.pgen.1007436 (PMC6010300; doi:10.1371/journal.pgen.1007436)

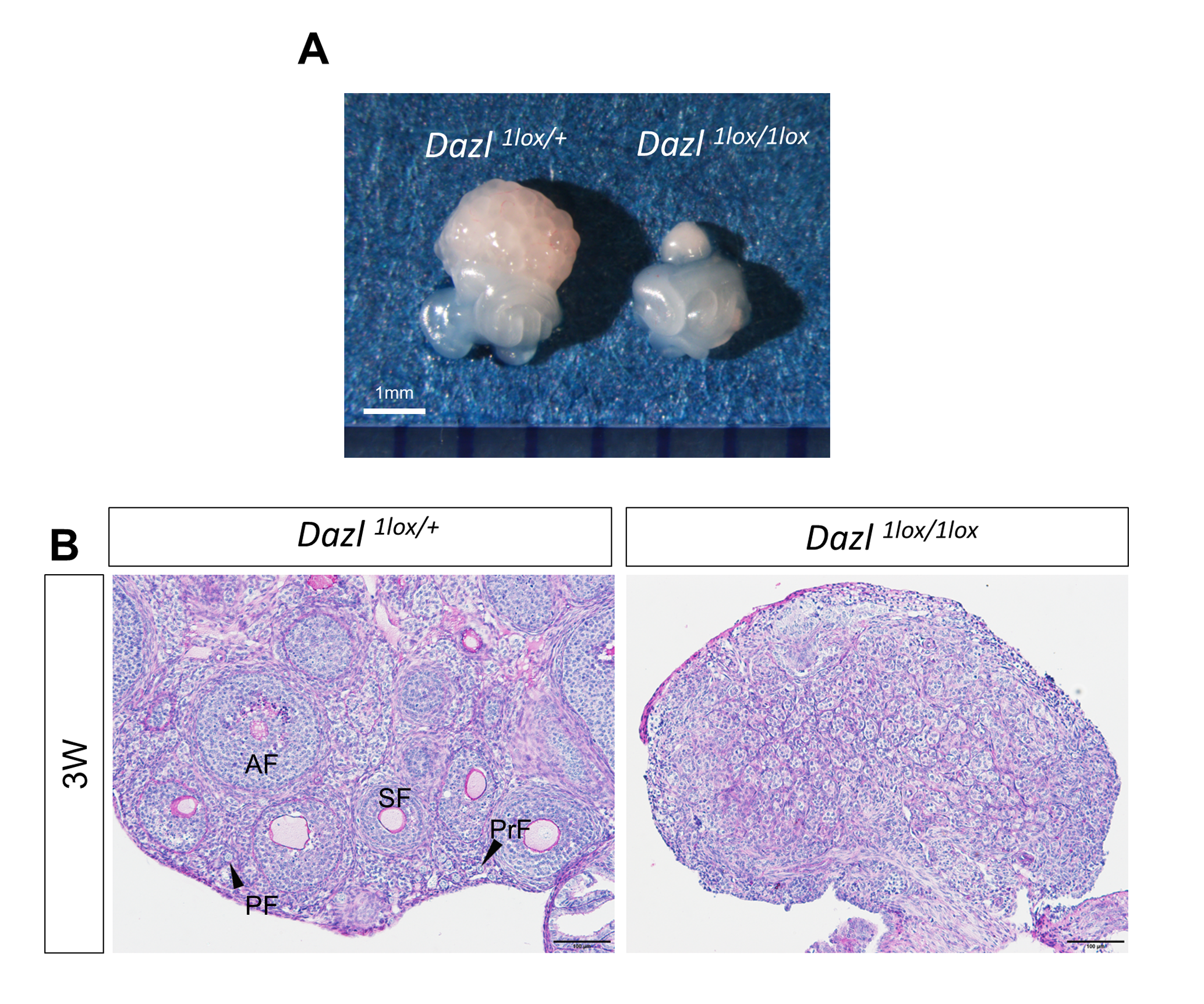

Supplement: S1 Fig — (A) A photograph of Dazl1lox/+ and Dazl1lox/1lox 3W ovaries. Scale bar, 1 mm. (B) PAS staining of Dazl1lox/+ and Dazl1lox/1lox 3W ovaries. Note that homozygous mutants contain no oocytes, reminiscent of the previous Dazl knockout ovary [16]. PrF, PF, SF, and AF are the same as in Fig 2C. Scale bar, 100 μm. (TIF) [file pgen.1007436.s001.tif]

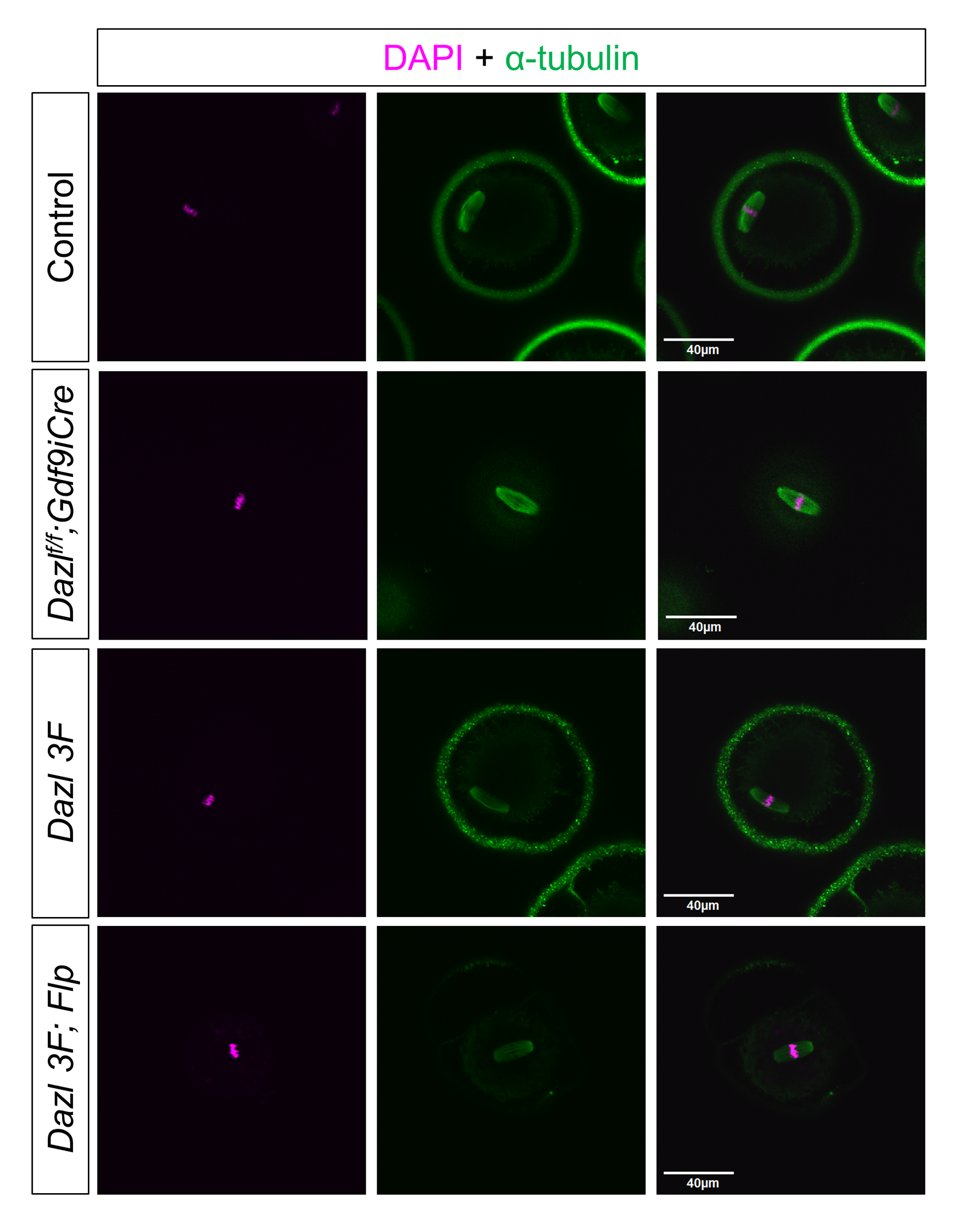

Supplement: S2 Fig — Immunostaining of MII oocytes of control (n = 85), Dazlf/f;Gdf9iCre (n = 34), Dazl 3F (n = 45) and Dazl 3F; Flp (n = 37) MII oocytes using an antibody against for α-tubulin (green). DNA was counterstained with DAPI (magenta). Scale bar, 40 μm. (TIF) [file pgen.1007436.s002.tif]

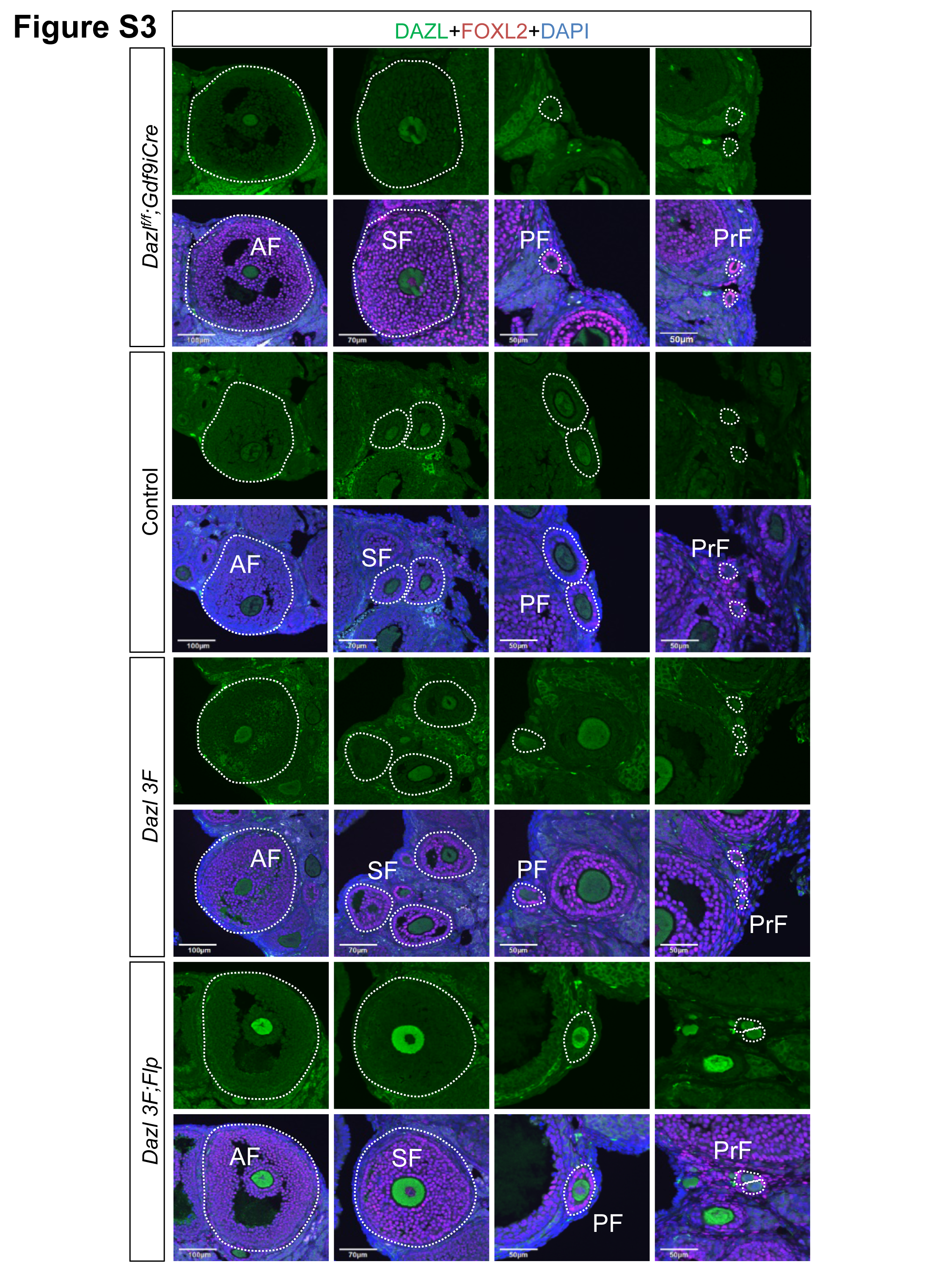

Supplement: S3 Fig — Immunostaining of 5W ovaries of control, Dazlf/f;Gdf9iCre, Dazl 3F and Dazl 3F; Flp mice. Antibodies against for DAZL (green) and FOXL2 (magenta) were used, and DNA was counterstained with DAPI. PrF, PF, SF, and AF are same as in Fig 2C. Scale bar, 50 μm. (TIF) [file pgen.1007436.s003.tif]

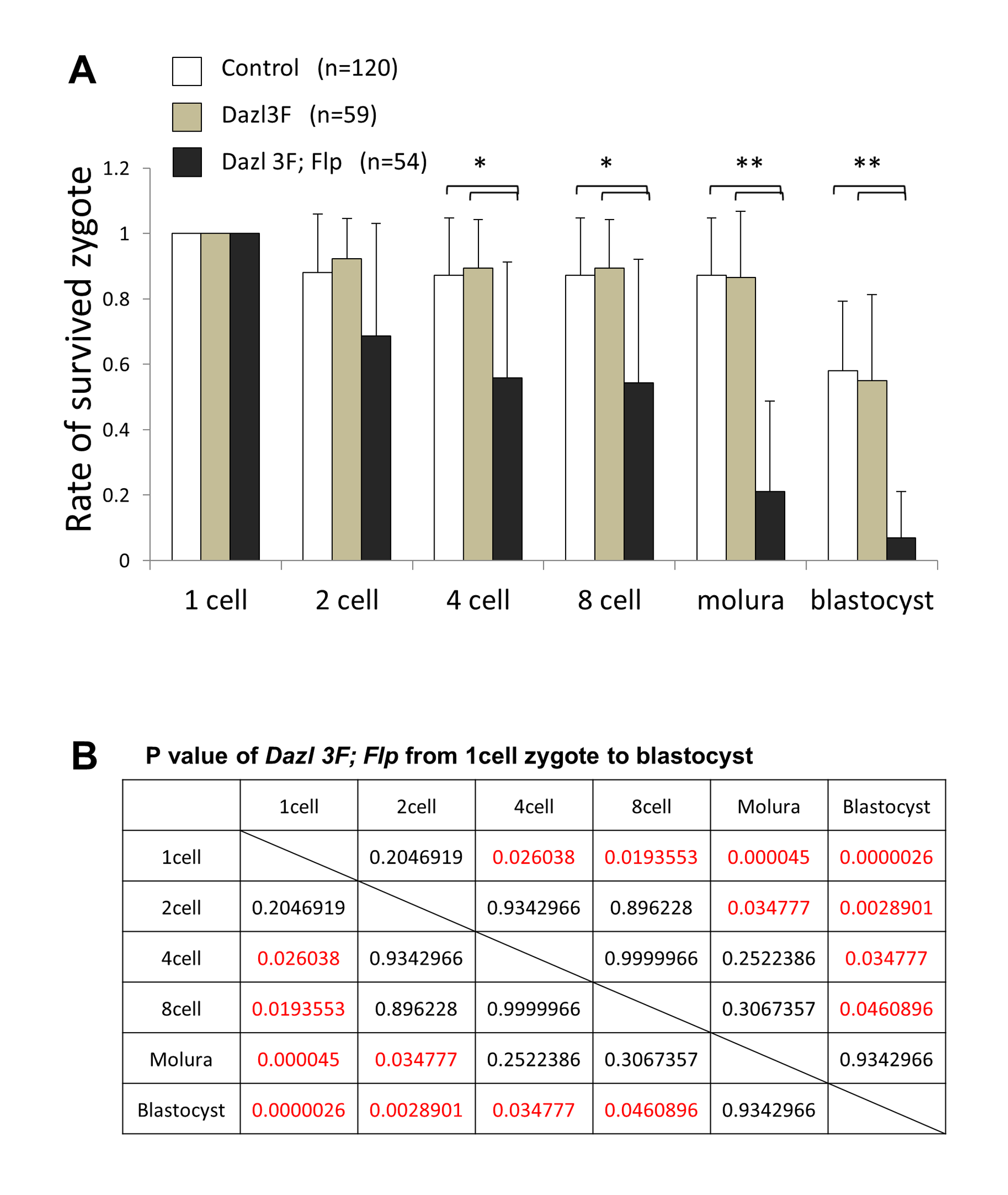

Supplement: S4 Fig — (A) Survival rates of preimplantation embryos. One-cell stage zygotes (n = total number of embryos examined, number of used mothers) were collected from control (n = 120, 15), Dazl 3F (n = 59, 7) and Dazl 3F; Flp (n = 54, 7) mothers. The proportion of surviving zygotes at each stage was calculated as follows: the number of surviving zygotes out of the number of 1-cell zygotes in each experiment (mother). Error bars, S.D. Significance level of changes are indicated (Tukey HSD; **P<0.005, *P<0.05). (B) Statistical analysis of surviving zygotes from 1-cell zygotes to blastocysts in Dazl 3F; Flp (n = 54). The p-value for the average number of zygotes in each stage was calculated using Tukey HSD. P<0.05 is written in red. (TIF) [file pgen.1007436.s004.tif]

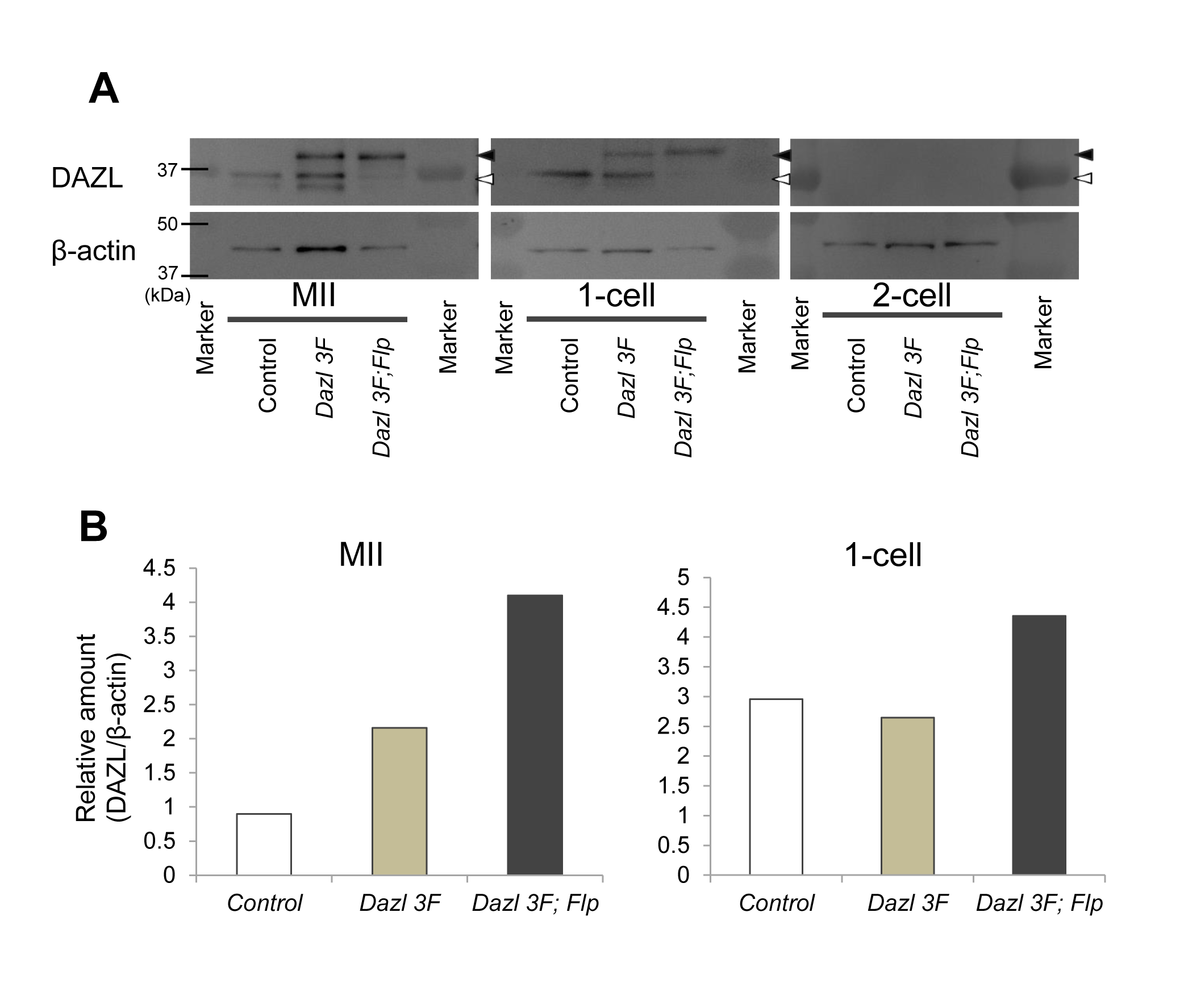

Supplement: S5 Fig — (A) Western blotting analysis of MII oocytes and 1- and 2-cell embryos. Both FLAG and endogenous DAZL were detected using the anti-DAZL antibody. Anti-β actin antibody was used as a loading control. Note that both FLAG and endogenous DAZL were not detectable in 2-cell embryos. Filled and open arrowheads indicate FLAG- and endogenous DAZL, respectively. (B) Quantification of western blotting results for MII and 1-cell samples. The vertical axis represents relative DAZL expression level normalized by β actin. (TIF) [file pgen.1007436.s005.tif]
